# Multi-Tone Harmonic Balance Optimization for High-Power Amplifiers through Coarse and Fine Models Based on X-Parameters

**DOI:** 10.3390/s22114305

**Published:** 2022-06-06

**Authors:** Lida Kouhalvandi, Osman Ceylan, Serdar Ozoguz, Ladislau Matekovits

**Affiliations:** 1 Department of Electrical and Electronics Engineering, Dogus University, Istanbul 34775, Turkey; lida.kouhalvandi@ieee.org; 2 Department of Electronics and Telecommunications, Politecnico di Torino, 10129 Turin, Italy; 3 Maury Microwave, Ontario, CA 91764, USA; osmanceylan@ieee.org; 4 Electronics and Communication Engineering Department, Faculty of Electrical and Electronics Engineering, Istanbul Technical University (ITU), Istanbul 34467, Turkey; ozoguz@itu.edu.tr; 5 Department of Measurements and Optical Electronics, Politehnica University Timisoara, 300006 Timisoara, Romania; 6 Istituto di Elettronica e di Ingegneria dell’Informazione e delle Telecomunicazioni, National Research Council, 10129 Turin, Italy

**Keywords:** automated design, coarse and fine modeling, deep neural network (DNN), harmonic balance (HB), high-power amplifier (HPA), multi-objective optimization, S-parameter, X-parameter

## Abstract

In this study, we focus on automated optimization design methodologies to concurrently trade off between power gain, output power, efficiency, and linearity specifications in radio frequency (RF) high-power amplifiers (HPAs) through deep neural networks (DNNs). The RF HPAs are highly nonlinear circuits where characterizing an accurate and desired amplitude and phase responses to improve the overall performance is not a straightforward process. For this case, we propose a *coarse and fine modeling* approach based on firstly modeling the involved transistor and then selecting the best configuration of HAP along with optimizing the involved input and output termination networks through DNNs. In the *fine phase*, we firstly construct the equivalent modeling of the GaN HEMT transistor by using X-parameters. Then in the *coarse phase*, we utilize hidden layers of the modeled transistor and replace the HPA’s DNN to model the behavior of the selected HPA by using S-parameters. If the suitable accuracy of HPA modeling is not achieved, the hyperparameters of the fine model are improved and re-evaluated in the HPA model. We call the optimization process coarse and fine modeling since the evaluation process is performed from S-parameters to X-parameters. This stage of optimization can ensure modeling the nonlinear HPA design that includes a high number of parameters in an effective way. Furthermore, for accelerating the optimization process, we use the classification DNN for selecting the best topology of HPA for modeling the most suitable configuration at the coarse phase. The proposed modeling strategy results in relatively highly accurate HPA designs that generate post-layouts automatically, where multi-tone harmonic balance specifications are optimized once together without any human interruptions. To validate the modeling approach and optimization process, a 10 W HPA is simulated and measured in the operational frequency band of 1.8 GHz to 2.2 GHz, i.e., the L-band. The measurement results demonstrate a drain efficiency higher than 54% and linear gain performance more than 12.5 dB, with better than 50 dBc adjacent channel power ratio (ACPR) after DPD.

## 1. Introduction

With the exceeding demand for faster and more reliable wireless communication systems, accurate models for high-power amplifier (HPA) designs that involve both active devices and passive components become increasingly essential [1]. The HPA plays a key role for conditioning the transmitted signals and consumes a large amount of power in system designs. Typically, realizing any radio frequency (RF) power amplifier (PA) starts with modeling of circuit components, and then continues with verification of the models with several measurements. The designers have to consider various topologies and techniques manually to achieve the desired design goals [2]. However, due to the increased complexity of the modern wideband communication systems, such as fifth generation (5G) and sixth generation (6G), intelligent design steps and advanced optimization methods are required [1,3].

Generally, *scattering parameters (S-parameters)* can be used for designing HPAs [4]. However, proper design and analysis of active circuits working with large signals require additional parameters that are dynamically linked to each other, such as amplitude of the input signal, input impedance, load impedance at fundamental and harmonic frequencies, biasing voltages/currents at the input and output, and temperature specifications [5,6]. Therefore, S-parameters should be extended to analyze nonlinear behavior of the circuit with a sufficient number of harmonics and adequate range of biasing. *X-parameters* are an extension form of S-parameters and can be extracted from large signal measurement data [7] or existing wideband nonlinear models. These parameters are based on the poly-harmonic distortion (PHD) and can be employed for modeling the nonlinear high-frequency components [5] and for designing amplifiers, as reported in [7,8,9,10]. Despite the fact that considerable research has been performed in this area, the usage of these factors to produce post-layouts is lacking. Hence, in this study, we construct our proposed optimization method based on X-parameters for providing comprehensive solutions.

After determining the proper modeling parameters, a suitable platform which incorporates different optimization schemes with X-parameters must be considered. Nowadays, popular optimizations such as Bayesian optimization, space mapping, genetic algorithm, and differential evolution [11] are not useful enough when the design parameters are in high dimension and advanced optimization techniques through machine learning and neural networks (NNs) for nonlinear designs are required substantially [12]. Constructing an accurate NN for these nonlinear circuits is not straightforward, and efficient methods are needed for finding the optimal hyperparameters of any NN (i.e., number of neurons and number of hidden layers) [13]. In [14], the usefulness of *space mapping* in the amplifier designs is proved; hence, this method can be a good solution for training and constructing NNs. The *coarse and fine modeling* method is a space mapping algorithm where the fine model achieves the optimal parameters without going to the direct optimization and uses the coarse model with updated values. It helps the designer to approximate and obtain accurate modeling in high-fidelity validations [15].

In this study, we present a superior experiment coarse and fine modeling approach based on multi-objective optimization algorithms where four various deep neural networks (DNNs) are employed, automatically. Due to the effective accuracy of DNNs (i.e., networks with more than two hidden layers), these types of NNs are used instead of shallow neural networks [16]. The proposed optimization method consists of two sequential phases for modeling: (i) active device (i.e., transistor), and (ii) HPA design, respectively. In the *“fine modeling”* phase, the gallium nitride (GaN) high-electron mobility transistor (HEMT) is modeled with X-parameters, and optimal hyperparameters that will be applied in the HPA modeling are achieved. Afterwards, three DNNs are developed for (i) selecting the best HPA configuration, (ii) modeling the selected HPA structure with S-parameters for re-evaluating the accuracy of achieved hyperparameters in the fine modeling phase (*“coarse modeling”* stage), and (iii) modeling the behavior of HPA by using multi-objective multi-verse optimizer (MOMVO) [17] for optimizing both one-tone and two-tone continuous wave (CW) harmonic balance (HB) performances, concurrently. By employing the multi-objective optimization, one-tone CW performances (i.e., output power ($P_{L}\left(\mathrm{dBm}\right)$), power gain ($G_{p}\left(\mathrm{dB}\right)$), and drain efficiency ($\eta_{D(\%)}$)) and also two-tone CW performances (includes harmonic and intermodulation distortions (IMDs (dBc))) are optimized once together [18,19].

In combination with [12,20], our proposed technique is demonstrated to be more effective for solving microwave modeling problems, and for optimizing multi-objective nonlinear HPA specifications jointly and automatically. Furthermore, by applying coarse and fine modeling method, higher-accuracy hidden layers’ structures are determined in an effective way. The HPA is modeled and optimized for nonlinear design specifications where not only one-tone performances are optimized but also two-tone performances up to 7th IMD are considered and optimized, concurrently. For accelerating the optimization process, the classification DNN is applied for selecting the well-matched HPA topology among various matching network (MN) configurations. The MOMVO algorithm is applied as it can beneficially approximate the Pareto-optimal front (POF) for more than three objectives in a cheaper computation [17]. At the final stage, for providing ready-to-fabricate layout, the constructed regression DNN with MOMVO method is applied. It optimizes the design parameters of the selected HPA model for achieving desired design specifications in conjunction with a full-wave electromagnetic (EM) analysis.

This article is organized as follows: Section 2 is devoted to describe the framework of the proposed optimization method including modeling of GaN HEMT transistor and HPA design optimizations through DNNs. Section 3 describes the practical implementation of proposed optimization method. Section 4 validates the proposed method by designing and measuring a 10 W HPA design. Lastly, conclusions are provided in Section 5.

## 2. Optimization-Oriented Strategy in a Nutshell

In microwave devices, optimizing nonlinear objective functions is not straightforward and needs powerful attempt. Employing NN with multi-layers (i.e., DNN) is a proficient technique for learning nonlinear behavior between input and output corresponding data [21]. Hence, in this section, we explain the framework of the proposed optimization method that leads to an automated HPA design with the aid of DNNs for improving output power, efficiency, gain, and linearity performances, concurrently.

The proposed optimization-oriented strategy is based on a “coarse and fine modeling” approach and it provides an automated optimization method for (i) modeling the active nonlinear GaN HEMT transistor, and (ii) optimizing the HPA’s performance in terms of one-tone and two-tone HB specifications, sequentially. In the first phase, the aimed transistor model (i.e., GaN model in this paper) is modeled with the X-parameters (fine modeling) using the “*regression DNN*”. After that, the “*classification DNN*” is employed for selecting the best configuration of HPA among various topologies achieved from the simplified real frequency technique (SRFT) [22]. Then, the third DNN (i.e., regression DNN) is used for modeling the selected HPA configuration, from the previous step, using the S-parameters (coarse modeling). For accelerating the optimization process, the hidden layer structure achieved from the fine modeling is used for constructing this third DNN and for verifying the accuracy of the trained network. This performance will accelerate the optimization process, leading to define the hyperparameters of HPA’s DNN, effectively. Finally, the last regression DNN (i.e., fourth DNN) is constructed, where the hyperparameters are the ones obtained from fine and coarse modeling. This network is employed for sizing the design parameters using multi-objective MOMVO algorithm where fabrication rules and constraints are also employed inside the optimization process. For polishing and achieving ready-to-fabricate layouts, various transmission line (TL)-microstrip models can be replaced, added to, or removed from the MNs of optimized HPA design.

Each NN to be constructed needs three kinds of data as training, validation, and testing data, defined as $X_{\mathrm{Train}}$, $X_{\mathrm{Val}}$, and $X_{\mathrm{Test}}$, with the division rate of 70%, 15%, and 15%, respectively. The corresponding responses of each data can also be provided as $Y_{\mathrm{Train}}$, $Y_{\mathrm{Val}}$, and $Y_{\mathrm{Test}}$. In all the presented networks, input layer features donate to $X_{\mathrm{Train}}$, $X_{\mathrm{Val}}$, and $X_{\mathrm{Test}}$ data and the output layer features yield to $Y_{\mathrm{Train}}$, $Y_{\mathrm{Val}}$, and $Y_{\mathrm{Test}}$ data. After generating suitable data, the NNs can be trained using (1). The accuracy of any NN is also measured with the difference amount between $Y_{\mathrm{Test}}$ and $Y_{\mathrm{Pred}}$, predicted outputs by $X_{\mathrm{Test}}$, as clarified in (2). These groups of data are achieved by arranging co-simulation environment between electronic design automation (EDA) tools (such as ADS) and numerical analyzer (such as MATLAB) [23] and by setting HB simulation environments in the ADS platform.

(1) $$\mathrm{net}=\mathrm{trainNetwork}(X_{\mathrm{Train}},Y_{\mathrm{Train}},\mathrm{layers},\mathrm{options})$$

(2) $$Y_{\mathrm{Pred}}=\mathrm{predict}\left(\mathrm{net},X_{\mathrm{Test}}\right)$$

**Figure 1:**
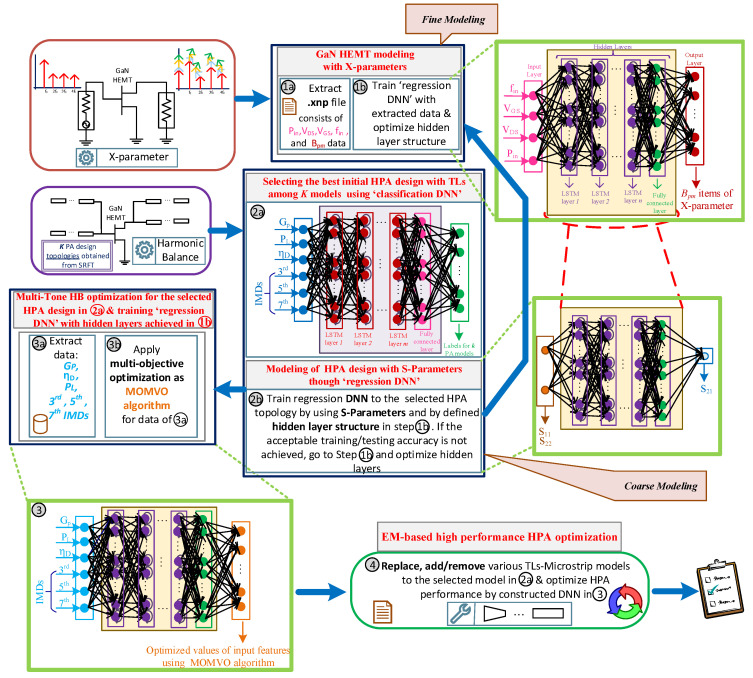
An overview of the proposed coarse and fine optimization method for modeling transistor and optimizing HPA designs with DNNs where multi-objective algorithm is employed.

In this work for the proposed classification DNN, softmax layer is used as an activation function and the crossentropyex layer is used as a loss function to the network with long short-term memory (LSTM) layers and one fully connected layer size of *k*. Additionally, for three regression DNNs, the rectified linear unit (ReLU) function is employed as the activation function, and the loss function is defined as the root mean squared error (RSME) along with LSTM layers. An overview of the automated proposed methodology is shown in Figure 1 and Algorithm 1 (at the end of this section).
**Algorithm 1:** Automated multi-tone HB optimization method**Initial preparation**:Prepare the co-simulation environment between the EDA tool and numerical analyzer;Determine the GaN HEMT transistor model;Provide the SRFT method on the numerical analyzer;Adjust one-tone and two-tone HB simulation setups in the EDA tool;Determine activation and loss functions for classification and regression DNNs;**Fine modeling through the regression DNN**:Insert the transistor model into the EDA environment and export X-parameters;Construct the regression DNN;(a)Determine the input layer features as: $f_{\mathrm{in}}$, $P_{\mathrm{in}}$, $V_{\mathrm{gs}}$, and $V_{\mathrm{ds}}$;(b)Calculate $B_{pm}$ (3);(c)Apply BO method for achieving initial hyperparameters;(d)Judgment: **If** required accuracy is not met, increase the number of layers, **Else** exit and go to the next step;**Prediction of best HPA configuration by the classification DNN**:Apply the SRFT method and achieve *K* different configurations;Construct the classification DNN;(a)Determine the input layer features as: $P_{L}$, $G_{p}$, $\eta_{D}$ and IMDs up to 7th order;(b)Define output layer features devoted to *K* models of various PAs;(c)Apply BO algorithm for achieving initial hyperparameters and train the network and train the network;(d)Judgment: **If** required accuracy is not met, increase the number of layers, **Else** exit and go to the next step;**Coarse modeling by the regression DNN**:(a)Pick the hidden layer structure from fine modeling;(b)Define input layer features ($S_{11}$ and $S_{22}$);(c)Determine output layer feature ($S_{21}$) and train the network;(d)Judgment: **If** required accuracy is not met, go to the fine modeling and re-optimize hidden layers, **Else** exit and go to the next step;**Design parameter optimization through MOMVO-based regression DNN**:(a)Envelop the fabrication rules and constrains;(b)Pick the hidden layer structure from fine/coarse modeling;(c)Define input layer features as: $P_{L}$, $G_{p}$, $\eta_{D}$ and IMDs up to 7th order;(d)Apply multi-objective MOMVO as a feature for output layer. Afterwards train and predict the optimal design parameters;**Polishing the final design structure**:(a)Add, remove, or replace the TLs with various TLs-microstrip models;(b)Prepare the ready-to-fabricate layout and save the design.

### 2.1. Modeling of GaN HEMT with X-Parameters (Fine Modeling)

Selecting a suitable semiconductor device with a wider bandgap plays a significant role in HPA designs. Hence, we prefer using GaN technology in this work due to its superior power density and durability that enable higher power operation at high frequencies in comparison with Si and GaAs devices [24]. Output spectrum of an active device such as GaN HEMT, operating in nonlinear region, carries not only the fundamental signal, but also the signals at harmonic frequencies. Hence, a considerable question is: How can this nonlinear device be modeled and characterized?

X-parameters are based on the poly-harmonic distortion and are used as an accurate and fast nonlinear modeling approach for representing small and large signal nonlinearity of S-parameters [7,8]. These harmonics consist of three terms: $X^{F}$, $X^{S}$, and $X^{T}$ in the output spectrum that are defined in (3) and (4). $X^{F}$ captures large signal harmonic response and $X^{S}$ with $X^{T}$ captures the small signal sensitivity by representing the incident and scattered waves. Functions for $B_{pm}$ (labeled with port *p* and harmonic *m*) are given small extraction tones as $A_{qn}$ (labeled with port *q* and harmonic *n*).

(3) $$B_{pm}=X_{pm}^{\left(F\right)}\left(\left|\begin{array}{c} A_{11} \end{array}\right|\right)P^{m}+X_{pm,qn}^{\left(S\right)}\left(\left|\begin{array}{c} A_{11} \end{array}\right|\right)P^{m-n}A_{qn}+X_{pm,qn}^{\left(T\right)}\left(\left|\begin{array}{c} A_{11} \end{array}\right|\right)P^{m+n}A_{qn}^{*}$$

where

(4) $$P=\frac{A_{11}}{\left|\begin{array}{c} A_{11} \end{array}\right|}$$

These X-parameters of active device (i.e., GaN HEMT in this study) can be extracted by preparing suitable simulation setup in EDA tool such as Keysight ADS [25]. For this case, firstly, four parameters, input frequency ($f_{\mathrm{in}}$), input power ($P_{\mathrm{in}}$), gate-source ($V_{\mathrm{gs}}$), and drain-source ($V_{\mathrm{ds}}$) voltage biasing are swept with appropriate step sizes. Afterwards, all the data appear in a file name such as *“.xnp”* (**Step**-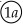). By using all the data and terms presented in the .xnp file and by constructing different output data (i.e., $B_{pm}$), the regression DNN presenting the active device model can be constructed. Input layer consists of inputs such as $f_{\mathrm{in}}$, $V_{\mathrm{GS}}$, $V_{\mathrm{DS}}$, and $P_{\mathrm{in}}$, where the output layer includes the X-parameters determined in (3) for *p* = 2 and *m* = 5.

To accurately train and construct the NN, a suitable and optimal set of hyperparameters (i.e., number of neurons and hidden layers) must be determined. For this case, we apply *Bayesian optimization (BO)* as it is well suited to optimize hyperparameters and it is faster than grid search and randomized search [26]. The BO aims to model hyperparameters that yield the lowest value of the score (i.e., error rate) by keeping Gaussian process (GP) model, internally. By applying BO, initial network hyperparameters for constructing DNN can be achieved (**Step**-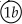).

### 2.2. Modeling and Optimizing HPA Design with DNNs Includes Coarse Modeling

In this section, we model the behavior of the HPA through DNNs for both one-tone and two-tone HB specifications. For this case, we firstly determine the best HPA configuration and topology using the classification DNN. Then, by using two other regression DNNs, the overall performance of HPA is optimized. Classification and regression DNNs can be constructed by defining input, hidden, and output layers with the activation and loss functions. The following is the explanation of each employed DNN and optimization process.

#### 2.2.1. Predicting Suitable HPA Configuration Using Classification DNN

The SRFT method is a suitable method for obtaining initial guess for the input and output MNs with distributed elements and design parameters for PAs. This method depends highly on the definition of ad hoc matrix, namely, $h_{i}$. The $h_{i}$ matrix is defined as [∓1, ∓1,…, ∓1]${}_{1\times t}$ where *t* ≥ 3 results in *k* various PA models differ in the number of TLs at input and output MNs. For selecting the most suitable topology and configuration among various *k* structures, classification DNN can be useful enough. By constructing this kind of DNN, any designer can define the desired output specifications and the DNN would predict which type (i.e., label) of PA can be appropriate among *k* various constructions. The following are the explanations for training the classification DNN.

##### A. Dataset Generation

Firstly, the selected GaN HEMT transistor model is inserted into the SRFT method that results in *k* different PAs, where these amplifiers vary in the number of TLs. Then an appropriate large amount of sampling dataset for training the classification DNN can be obtained by employing Gaussian random distribution with 5% of standard deviation (σ) around the achieved component values from the SRFT method, which corresponds to 15% of 3.σ deviation. For *k* different amplifier configurations, *p* data in terms of input layer features are obtained. Hence, *k*×*p* data including $X_{\mathrm{Train}}$, $X_{\mathrm{Val}}$, and $X_{\mathrm{Test}}$ can be generated.

##### B. Features of Input, Hidden, and Output Layers for Classification DNN

As the classification DNN in **Step**-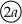 shows, the features of input layer consist of one-tone HB specifications such as ($P_{L}$, $G_{p}$, $\eta_{D}$), and two-tone specifications such as (third, fifth, seventh IMDs).

**Figure 2:**
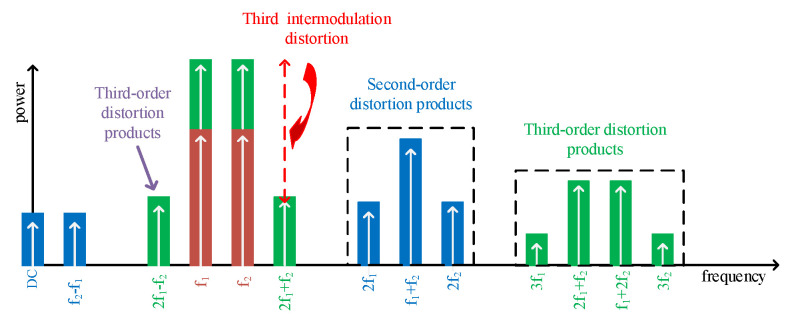
Third-order intermodulation distortion and products.

For obtaining $P_{L}$, $G_{p}$, and $\eta {}_{D}$ specifications, a *single-tone HB* simulation environment, and for achieving IMDs (i.e., ${\mathrm{IMD}}_{3}$, ${\mathrm{IMD}}_{5}$, and ${\mathrm{IMD}}_{7}$), a *two-tone HB* simulation environment must be set, where various values for each frequency appear. Even-order IMDs such as second, forth, and so on appear inside the input tones; however, odd-order tones such as third, fifth, and so on interface with the fundamental tones, and these interruptions can make problems for RF designs. For illustrating the ${\mathrm{IMD}}_{3}$ characteristic, Figure 2 shows third-order distortion products in a wide range of frequencies where there are certain mixing tones and distortion products for the ${\mathrm{IMD}}_{3}$. The extended version of Figure 2 for the fifth and seventh IMDs can be developed in special tones, and the following is the summary of distortion products for different tones.

The first tone mix is [{1,0}, {0,1}]. The third, fifth, and seventh order distortion orders are [{2,−1},{1,0},{0,1},{−1,2}], [{3,−2},{2,−1},{1,0},{0,1},{−1,2},{−2,3}], and [{4,−3},{3,−2},{2,−1},{1,0},{0,1},{−1,2}], [{−2,3},{−3,4}], sequentially. All of these data are presented in a file named *“spectra.raw”* in the arranged co-simulation environment and all the data are in the format of voltage and current. Hence, by using MATLAB tool and defining the especial expressions, the aimed specifications can be calculated.

Each set of *p* data is denoted by “1”,“2”,…,“*k*” categorical labels or numeric responses for presenting the output layer features (i.e., $Y_{\mathrm{Train}}$, $Y_{\mathrm{Val}}$, and $Y_{\mathrm{Test}}$). For this DNN, the initial number of LSTM layers with neuron numbers is estimated using the BO algorithm.

#### 2.2.2. Coarse Modeling

HPAs are nonlinear circuits that include a large number of design parameters. For this case, modeling of this kind of design is not straightforward and requires serious attention. For our problem, coarse modeling can be performed by training the regression DNN through S-parameters, which are the simplified version of X-parameters. This type of modeling can help designers in finding the optimal hyperparameters of regression DNNs with reduced difficulty for designing HPAs. For this third DNN, the features of input layer are $S_{11}$ and $S_{22}$ and the feature of output layer is $S_{21}$. The sampling data for this network can be generated by iterating the component values of selected HPA design in (**Step**-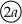) in the range of ∓5%, ∓10%, ∓15%, ∓20%, and ∓25%.

In this phase, the optimal hyperparameters from the “fine modeling” step are used to model the selected HPA configuration through the classification DNN. If the trained DNN does not respond with acceptable accuracy, the fine model’s hyperparameters are changed and re-evaluated in the coarse modeling phase until the desired accuracy is achieved (i.e., more than 90%) (**Step**-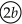).

#### 2.2.3. Optimizing Design Parameters with Multi-Objective-Based Regression DNN

After achieving the optimal hyperparameters of regression DNN from coarse modeling, it is time to predict the optimal design parameters of the selected topology. **Step**-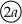 results in high-performance one-tone and two-tone specifications, concurrently. Here, the regression DNN is trained where the hyperparamers are the ones achieved from the fine and coarse modeling phases (**Step**-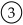). In this step, it is targeted to optimize HPAs that can pass the EM-based simulations, resulting in generating ready-to-fabricate layouts. Hence, some design rules and fabrication criteria must be employed inside the optimization process, as Table 1 clarifies. The following also presents the features of input layer and output layer, and also the method of generating data for training the fourth regression DNN.

##### A. Features of Input, Output, and Hidden Layers of the Fourth DNN

The input layer features of the last regression DNN are $\eta {}_{D}$, $P_{L}$, $G_{P}$, and IMDs. The approach for extracting these specifications is described in Section 2.2.1. The procedure of generating the output layer feature is as follows:

The aim of our optimization method is to enhance efficiency, output power, power gain, and also linearity concurrently for the HPA designs. For this case, the single objective function must be constructed using the weighted sum of ${\mathrm{IMD}}_{3}$, ${\mathrm{IMD}}_{5}$, ${\mathrm{IMD}}_{7}$, $G_{P}$, $P_{L}$, and $\eta {}_{D}$ (**Step**-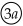). These specifications are heterogeneous functions and should be normalized before combining into a single objective function. Hence, the objective function can be defined as (5), where each metric is normalized using their respective mean value and standard deviation estimated from the values located around the neighborhood of the optimum Pareto point.

(5) $$F_{obj}=w_{1}\left[\begin{array}{c} \frac{{\mathrm{IMD}}_{3}-\bar{{\mathrm{IMD}}_{3}}}{\sigma_{{\mathrm{IMD}}_{3}}} \end{array}\right]+w_{2}\left[\begin{array}{c} \frac{{\mathrm{IMD}}_{5}-\bar{{\mathrm{IMD}}_{5}}}{\sigma_{{\mathrm{IMD}}_{5}}} \end{array}\right]+w_{3}\left[\begin{array}{c} \frac{{\mathrm{IMD}}_{7}-\bar{{\mathrm{IMD}}_{7}}}{\sigma_{{\mathrm{IMD}}_{7}}} \end{array}\right]+w_{4}\left[\begin{array}{c} \frac{G_{p}-\bar{G_{p}}}{\sigma_{G_{p}}} \end{array}\right]+w_{5}\left[\begin{array}{c} \frac{P_{L}-\bar{P_{L}}}{\sigma_{P_{L}}} \end{array}\right]+\log\left({\left[\begin{array}{c} \frac{\eta_{D}-\bar{\eta_{D}}}{\sigma (\eta_{D})} \end{array}\right]}^{w_{6}}\right)$$

For optimizing the determined multi-objective function, we use the MOMVO algorithm for obtaining optimal solutions, which is based on evolutionary algorithms. This method as a multi-objective optimization is preferred to other well-known methods, such as multi-objective particle swarm optimization [27], multi-objective evolutionary algorithm based on decomposition [28], gravitational search algorithm [29], and gray wolf optimizer [30] due to its beneficial solutions.

##### B. Data Generation for Training the Fourth DNN

After determining the various features of DNN layers, it is time to introduce how the sampling data are generated for training and constructing the final regression DNN. For this case, we prepare sampling data summarized in (6), where *k* denotes number of various PA models with TLs achieved from the SRFT method and *j* represents the iteration of component values randomly for each PA model within the range of [∓5%–∓50%] with step size of 5%. By sweeping the sampling design parameters, multi-tone HB results are optimized by the MOMVO algorithm and are exerted to the output layer (**Step**-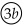).

(6) $$\sum_{i=1}^{k}\sum_{j}^{}\left(G_{p_{i,j}},\eta_{D_{i,j}},P_{L_{i,j}},IMD_{3_{i,j}},IMD_{5_{i,j}},IMD_{7_{i,j}}\right)$$

### 2.3. Electromagnetic-Based HPA Design with TLs

After modeling and optimizing the HPA design, the post-layout must be generated for fabrication. With the target of polishment to the final layout, any designer can remodel the TLs of the optimized HAP in **Step**-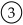 with other *TLines microstrip library palettes* existing in the ADS platform (**Step**-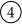). This step helps the designers to generate more reliable layouts that have passed the EM simulations and are more suitable schemes for fabrication in the RF companies.

## 3. Practical Implementation of Coarse and Fine Modeling Approach

Our proposed automated optimization method is exerted on the CPU execution environment, featuring an Intel Core i7-4790 CPU @ 3.60 GHz with 32.0 GB RAM. In this work, we use Ampleon CLF1G0060-10 GaN HEMT as the dynamic load-line model of this transistor, which has been verified in [20]. The transistor is modeled by the regression DNN that is based on X-parameters by obtaining boundary knowledge of $f_{\mathrm{in}}$, $P_{\mathrm{in}}$, $V_{\mathrm{gs}}$ and $V_{\mathrm{ds}}$ from the data sheet of the transistor and by generating the $B_{pm}$ items that are described in (3) and (4).

**Figure 3:**
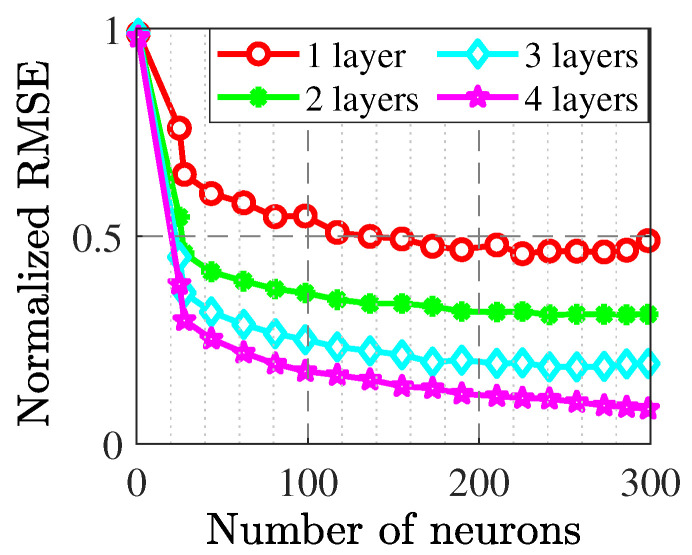
Sequence of achieving accurate number of hidden layers in modeling GaN HEMT transistor.

The fine modeling phase starts with one hidden layer, and the number of neurons are predicted using the BO method. Figure 3 shows the normalized RMSE in one hidden layer that is around 0.5 and is not acceptable. Hence, sequentially, the number of hidden layers are increased such that in the fourth layer with 300 neurons, the normalized RMSE is decreased to 0.07. The total sampling data contains 1700 sequences in 200 sweeping points and includes multi-segment output results.

After modeling the GaN HEMT, it is time to predict the best HPA topology and configuration that fits best to the used transistor model. For this case, the classification DNN is employed. In order to achieve various topologies and configurations, the SRFT method is employed, resulting in 11 different amplifier models that differ in the number of TLs. For each model, 500 sequences, including multi-segment one-tone and two-tone CW values (i.e., $G_{p}$, $\eta {}_{D}$, $P_{L}$, ${\mathrm{IMD}}_{3}$, ${\mathrm{IMD}}_{5}$, and ${\mathrm{IMD}}_{7}$), over the operation bandwidth are prepared, where in total, 500×11=5500 data are generated. The hyperparameters of the classification DNN are predicted using the BO method, where, in the fifth LSTM layer with 350 neurons, it obtains the testing accuracy of 98.2%.

**Figure 4:**
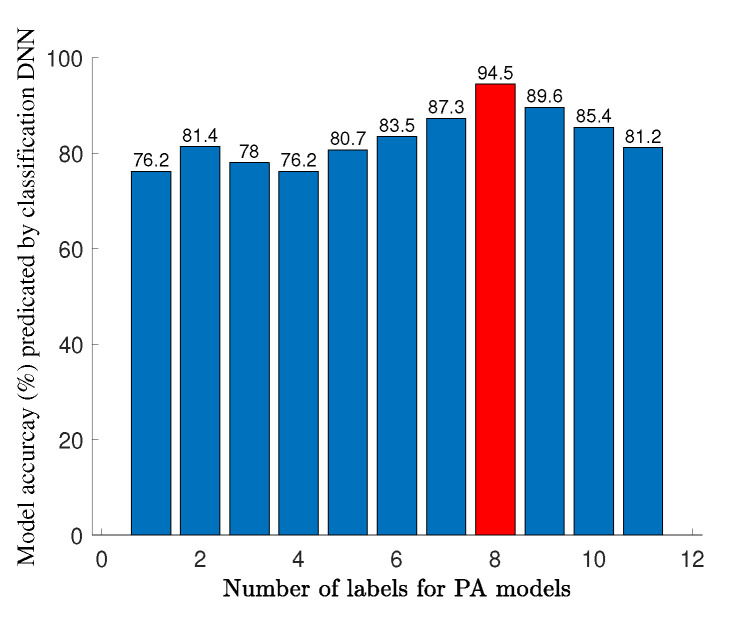
Accuracy prediction of 11 models using the classification DNN.

After constructing the classification DNN, the determined one-tone and two-tone features of the used transistor model are entered to the DNN. The trained DNN predicts that the eighth amplifier model achieved from the SRFT method can be a suitable topology and structure to start designing (see Figure 4).

Afterwards, the coarse modeling including the regression DNN is applied and the hyperparameters are taken directly from the fine modeling that involves four LSTM layers and 300 neurons in each hidden layer. The input layer and output layer of this DNN include S-parameters $S_{11}$, $S_{22}$, and $S_{21}$, respectively. The accuracy of this DNN (i.e., third network) is significant since it paves the way of constructing fourth DNN for sizing the design parameters. In simple words, modeling the HPA design with S-parameters can make the designer sure of the accuracy of the constructed DNN.

The last regression DNN is constructed for sizing the design parameters of the selected topology (i.e., eighth amplifier model from SRFT method). For this case, easily, the hyperparameters are taken from coarse modeling, and input layer and output layer features are constructed. As shown in Figure 1, the input layer consists of $P_{L}$, $G_{p}$, $\eta_{D}$, and IMDs, where the output layer consists of the optimized values of these input features using the MOMVO algorithm (see Equation (5)). It must be noted that in this phase, design rules are also applied for preparing the layout that can pass the EM simulation. The total sampling data for this regression DNN is around 10,000, which includes multi-segment input layer features over the operational frequency band. Finally, the polishing process is employed for finding the appropriate TL microstrip model among various existing libraries in the ADS tool and also for generating a post-layout that passes the EM simulation and is ready to be fabricated in the companies.

## 4. Fabrication and Measurement of Optimized HPA

The proposed optimization method using vendor component models is applied for optimizing the design of a 10 W HPA consisting of a CLF1G0060-10 GaN HEMT transistor. This transistor model is a 6 GHz general-purpose RF power transistor that is without any in-package matching network, and its wideband verified nonlinear model is provided by the manufacturer. The targeted HPA design is optimized for enhancing efficiency, power gain, output power, and linearity in the operational frequency band of 1.8 GHZ to 2.2 GHz (i.e., cellular frequency band used in mobile wireless communications).

**Figure 5:**
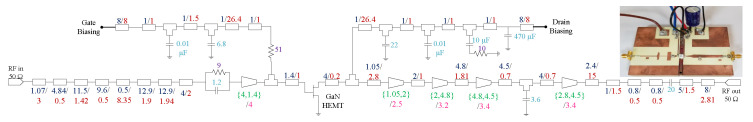
Fabricated EM-based HPA, designed by proposed DNN-based optimization method. Units of each capacitor and resistor are pF and Ohm, respectively; “/” represents width/length in mm unit and “$\left\{\begin{array}{c} , \end{array}\right\}$” represents $Width_{1},Width_{2}$ of tapered lines in mm unit.

The optimized HPA is biased at a drain voltage of 50 V with a quiescent drain current of 40 mA and fabricated on a Rogers RO4350B substrate with $\varepsilon_{r}$ = 3.66 and a thickness of 0.508 mm. The final HPA layout with design parameters is depicted in Figure 5. The implemented HPA’s performance is measured with CW signals using the Maury Microwave’s MT2000 mixed-signal characterization system. The measurement setup enables the large signal measurements with frequency and power sweep using single-tone, two-tone, and modulated signals.

**Figure 6:**
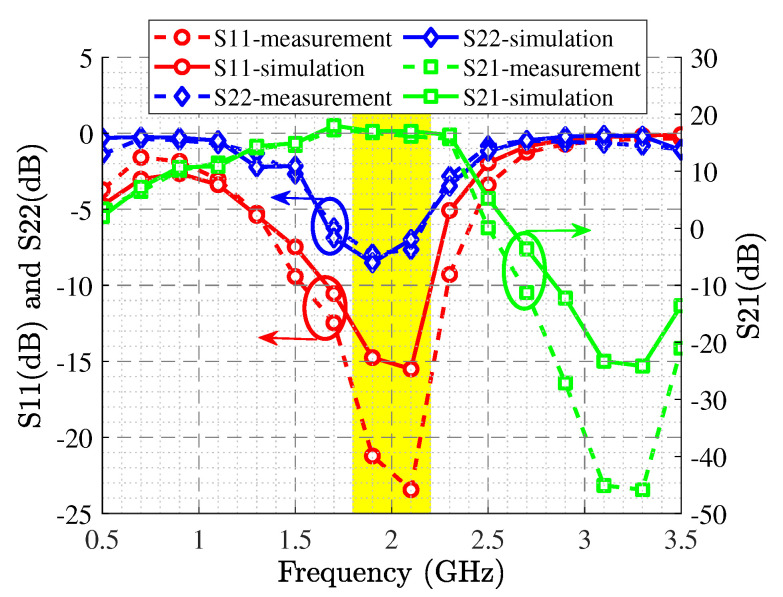
Simulated and measured S-parameters of the optimized HPA.

**Figure 7:**
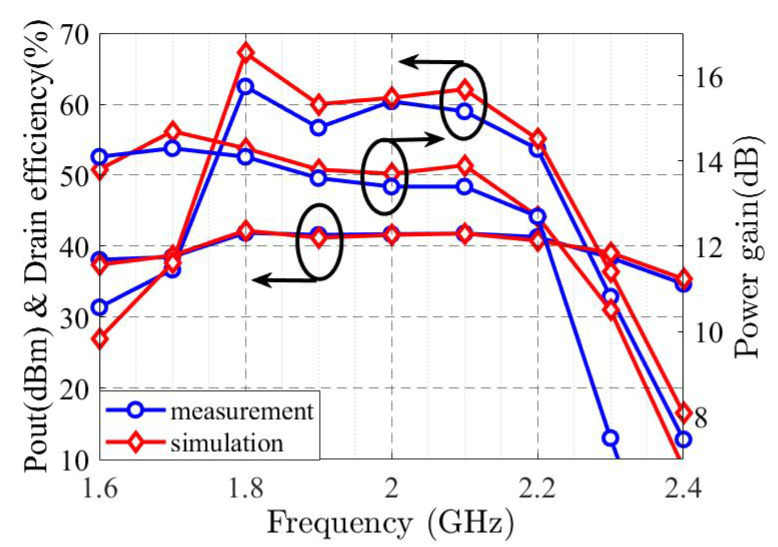
One-tone CW simulated and measured output power, drain efficiency, and power gain at 3 dB gain compression.

**Figure 8:**
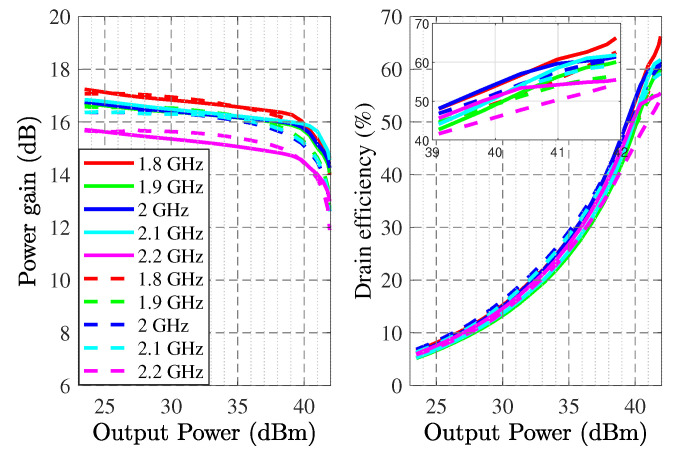
Power gain and drain efficiency over output power simulated (dash lines) and measured (solid lines) at various frequencies.

The wideband S-parameter simulation and measurement results are shown in Figure 6, and it illustrates the $S_{21}$ larger than 15.5 dB and the $S_{11}$ lower than −16 dB in the operational frequency band. The $G_{p}$, $\eta_{D}$, and $P_{L}$ specifications are displayed in Figure 7, revealing the $G_{p}$ between 12.5 dB and 14.1 dB, $\eta_{D}$ higher than 54%, and around 41 dBm $P_{L}$ at p3dB. Figure 8 shows the simulated and measurement $G_{p}$ and $\eta_{D}$ variation with the increasing output power at the center and corner frequencies.

**Figure 9:**
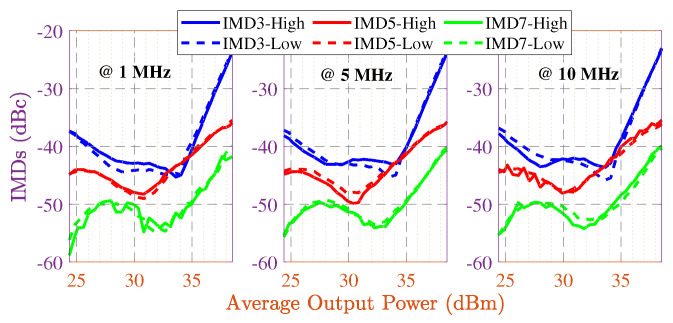
Measured high and low IMDs with 1 MHz, 5 MHz, and 10 MHz tone spacing values.

**Figure 10:**
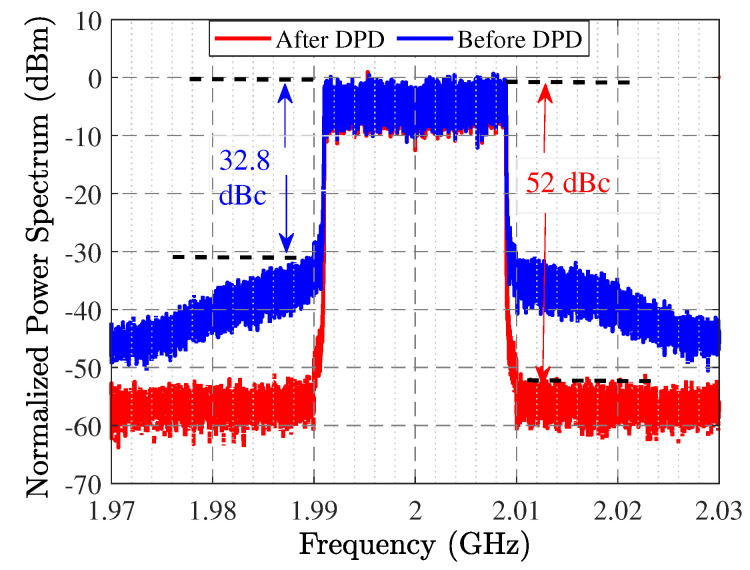
Measured output spectrum with 20 MHz LTE signal having 10.7 dB PAPR, with and without DPD.

High and low third, fifth, and seventh inter-modulation products (i.e., ${\mathrm{IMD}}_{3}$, ${\mathrm{IMD}}_{5}$, and ${\mathrm{IMD}}_{7}$) are depicted in Figure 9. LTE supports six different signal bandwidth options: 1.4 MHz, 3 MHz, 5 MHz, 10 MHz, 15 MHz, and 20 MHz. We have tested the designed amplifier with the widest bandwidth signal of the LTE standard, 20 MHz, to evaluate its linearity. Figure 10 presents the modulated signal response of the optimized HPA design with and without digital predistortion (DPD). In the measurement stage, a 20 MHz LTE signal with 10.7 dB peak-to-average power ratio (PAPR) is used for the analysis. For our optimized HPA, better than −50 dBc adjacent channel power ratio (ACPR) is achieved at around 32.1 dBm average output power.

As mentioned above, the HPAs being nonlinear circuits during their design, various parameters must be considered. Table 2 presents the state-of-the-art situation on the employed optimization algorithms and methodologies for designing amplifiers. Our proposed method is concurrently optimizing various design specifications such as efficiency, output power, power gain, and various IMDs, automatically, while in the recently published literature, optimization is limited to a lower number of specifications.

## 5. Conclusions

In this work, we present an automated optimization process for solving high-dimensional modeling problems of HPA designs. In our technique, we apply coarse and fine modeling by using four DNNs based on transistor modeling with X-parameters (fine model), classification DNN, coarse modeling, and, finally, multi-objective optimization method for sizing the design parameters. The proposed method provides an advanced high-accuracy multi-objective optimization process for training data in a high-dimensional space with optimal hyperparameters. For validating the proposed multi-tone optimization method, we design and fabricate a 10 W HPA design; the results reveal high-performance outcomes in terms of power gain, efficiency, output power, and linearity.

The achieved results show that despite the complexity of the goals and large amount of the nonlinear parameters to be optimized, automated design techniques and optimization algorithms can be applied to nonlinear RF circuit design with regular workstations. Cross-relations of the optimization methods and device models have key roles for performance of automated RF circuit designs. Future studies can be focused on developing and integrating objective functions, considering the weighting algorithms and device model interpolations.

## Figures and Tables

**Table 1:** Fabrication and design rules for layout and components.

Design Rule	Value
Width	⩾0.5 mm
Width for biasing lines	⩾1 mm
Length	⩽30 mm
Capacitor	⩾0.2 pF
Resistor for RF lines	⩾5 Ω
Length for T junctions	⩾0.5 mm
Min spacing between the lines	⩾0.5 mm

**Table 2:** Summary of various methodologies and the optimization goals.

Ref.	Method	Optimization Goals
[31]	Real frequency technique	- Efficiency
[32]	Simplified real frequency technique	- Efficiency;
		- Fractional bandwidth
[33]	Bayesian optimization	- Efficiency
[34]	Shot-stepped Chebyshev impedance transformers	- Bandwidth
[35]	Systematic approach based on source and load pull	- Efficiency;
		- Fractional bandwidth
[36]	Bounded performance technique	- Power;
		- Fractional bandwidth
[37]	Genetic algorithm	- Fractional bandwidth
[20]	Classification and TSEMO-based regression DNNs	- Efficiency
		- Output power
		- Power gain
This work	Coarse and fine modeling with DNNs based on X-parameters	- Efficiency
		- Output power
		- Power gain
		- ${\mathrm{IMD}}_{3}$, ${\mathrm{IMD}}_{5}$, and ${\mathrm{IMD}}_{7}$

## Data Availability

Not applicable.
